# Transcatheter tricuspid valve intervention techniques and procedural steps for the treatment of tricuspid regurgitation: a review of the literature

**DOI:** 10.1136/openhrt-2022-002030

**Published:** 2022-06-01

**Authors:** Kamal Matli, Ahmad Mahdi, Victor Zibara, Christy Costanian, Georges Ghanem

**Affiliations:** 1 Cardiology, Lebanese American University Medical Center-Rizk Hospital, Beirut, Lebanon; 2 Cardiologie, Centre Hospitalier de Haguenau, Haguenau, Alsace-Champagne-Ardenne-Lorraine, France; 3 LAU Gilbert and Rose-Marie Chagoury School of Medicine, Byblos, Lebanon

**Keywords:** Transcatheter Tricuspid Valve Interventions, Tricuspid Regurgitation, Procedural Steps, Percutaneous Interventions, Annuloplasty, Coaptation device, CAVI

## Abstract

Severe tricuspid regurgitation (TR) is an undertreated common pathology associated with significant morbidity and mortality. Classically, surgical repair or valve replacement were the only therapeutic options and are associated with up to 10% postprocedural mortality. Transcatheter tricuspid valve interventions are a novel and effective therapeutic option for the treatment of significant TR. Several devices have been developed with different mechanisms of action. They are classified as annuloplasty devices, replacement devices, caval valve implantation and coaptation devices. In this review, we provide a step-by-step description of the procedural steps and techniques of every device along with video support.

## Introduction

Significant tricuspid regurgitation (TR) has been associated with poor long-term outcomes that include but are not limited to right heart failure, end-organ damage such as liver failure, and mortality.^1 2^ Moderate/severe TR has also been associated with an increase in all-cause mortality independent of left ventricular ejection fraction, pulmonary pressures and right ventricle (RV) dysfunction. TR is classified into primary or secondary TR.^3 4^ Primary TR, present in 8%–10% of TR cases, is due to an isolated congenital or acquired pathology of the tricuspid valve (TV) or subvalvular apparatus. Aetiologies are numerous and include the presence of a device lead passing through the valve, systemic diseases, endocarditis, radiation, tumours, along other causes.^3 5^ Secondary or functional, the most common form of TR accounting to more than 90% of the cases, is usually secondary to left-sided heart failure or atrial fibrillation. Functional TR is mainly caused by annular dilation due to RV-free wall dilation, and leaflet tethering due to lateral and apical displacement of the papillary muscle.^6–11^

Significant TR presents with signs and symptoms of right-side heart failure. These include ascites, fatigue, peripheral oedema, painful hepatosplenomegaly and bloating.^12^ When left untreated, TR is associated with a rescued survival mainly due to hepatic and/or renal failure.^1^

The disease currently has limited treatment options that are challenging, themselves. The management of TR includes medical, surgical and percutaneous interventional treatments. Medical therapy for the management of severe TR is often limited and mainly targets symptom relief. Furthermore, surgical replacement or repair is associated with a high rate of complications and an in-hospital mortality reaching up to 10% especially in patients with previous left-sided heart valve surgery or previous tricuspid repair.^13–16^

As such, a novel modality for the treatment of isolated TR was developed in the form of transcatheter tricuspid valve intervention (TTVI). TTVI is classified according to the mechanism of action as such: annuloplasty devices, replacement devices, caval valve implantation (CAVI) and coaptation devices.^17^ We aim to provide an updated overview of the current evidence concerning TTVI, focusing on the main procedural steps and future directions of the field.

### Anatomy

A normal TV complex includes a fibrous annulus, usually three leaflets, papillary muscles, chordae tendinae, right ventricular myocardium and a right atrial myocardium.^18 19^ It is the most anterior valve, and it is located between the right atrium (RA) and RV. The TV is the largest cardiac valve and has an area that ranges between 7 and 9 cm^2^. The tricuspid annulus (TA), on the other hand, is an asymmetric and dynamic saddle-shaped ellipsoid, which allows it to change with varying loading conditions. Its circumference is 12±1 cm and its area is 11±2 cm^2^.^20 21^ The TV is a simple three-leaflet valve: the anterior leaflet that extends from the anteroseptal commissure until the anterior papillary muscle, the posterior leaflet that extends from the anterior papillary muscle along the inferior wall of the RV to the posteroseptal commissure and the septal leaflet that is attached to the interventricular septum.^18 19 22 23^ However, its anatomy is more complex and variable. Some recent studies show that it can present as a bicuspid or a quadricuspid valve.^24 25^ Analysis of 579 patients in 1 multinational retrospective study showed that our valve of interest has 3 well-defined leaflets in only approximately 54% of patients, while it has 4 functional leaflets in approximately 39% of patients, had 2 leaflet configuration in 4.5% of patients and had 5 leaflet configuration in 2.4% of patients.^26^ Anatomic variations of the TV may occur in the context of other congenital anomalies and syndromes. In addition, the number, shape and length of the chordae tendineae and papillary muscles vary.^27^

A detailed study of the TV anatomy, especially the location and number of supernumerary leaflets or scallops, is primordial for procedural planning and may affect intervention outcome.

### Selection criteria

**Figure 1:**
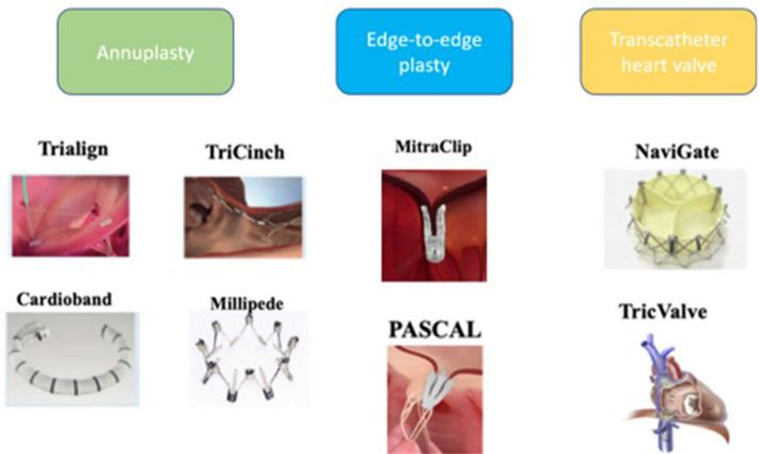
Tricuspid valve intervention devices with reported clinical use from Advances in transcatheter mitral and tricuspid therapies’ by Overtchouk et al.,2020, B*MC Cardiovascular Disorders* 20, 1. Reprinted with permission from Overtchouk *et al*.^61^

**Figure 2:**
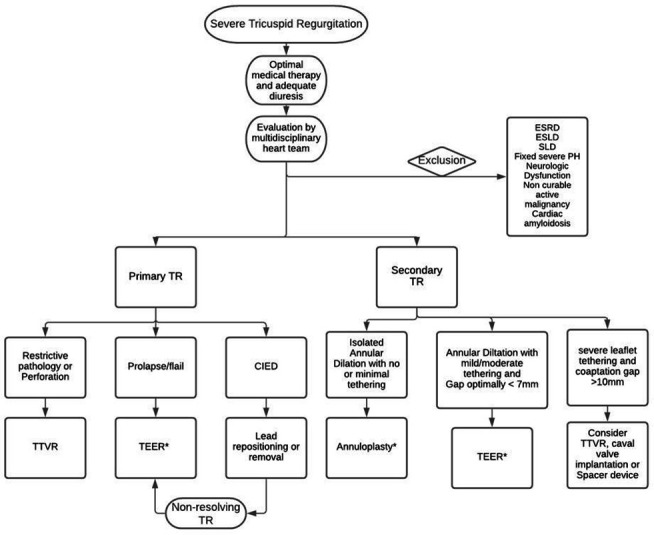
Proposed algorithm to guide transcatheter tricuspid valve intervention device selection. CIED, cardiovascular implantable electronic device; ESLD, end-stage liverg disease; ESRD, end-stage renal disease; PH, pulmonary hypertension; SLD, severe lung disease; TEER, transcatheter edge to edge repair; TR, tricuspid regurgitation; TTVR, transcatheter tricuspid valve replacement. *: refer to table 2 for ideal and complicating factors of technique.

Patient selection for TTVI should be done by a multidisciplinary heart team to assess the chances of procedural success and clinical benefit. After maximising medical therapy, especially diuresis, the cause and mechanism of TR in addition to right ventricular function and size must be evaluated.^28^ Surgery has classically been considered the first-line treatment, however, if after evaluation, the patient is deemed inoperable or at high surgical risk it was demonstrated that TTVI was associated with a lower all-cause mortality and rehospitalisation when compared with optimal medical therapy alone.^29^ Randomised controlled trials are needed to expand further on the clinical efficacy and optimal selection criteria of such interventions. Currently, selecting the appropriate technique and device can be difficult since there are several devices present on the market(figure 1) with the absence of head-to-head clinical trials to compare their outcomes. Thus, the choice of the device must be based on anatomic and physiologic features. Principle device characteristics are presented in table 1. A proposed algorithm to guide device selection based on the mechanism and characteristics of the regurgitant lesions is provided in figure 2. Furthermore, table 2 provides ideal and complicating factors for TTVI.

**Table 1:** Transcatheter tricuspid valve device technical characteristics^62^

Device	Technique	Access	Sheath (Fr)	Procedural success	Study
TriCinch	Annuloplasty	TF	24	75%	PREVENT
Trialign	Annuloplasty	TJ	14	93%	SCOUT I
Cardioband	Annuloplasty	TF	24	100%	TRI-REPAIR
TRAIPTA	Annuloplasty	TF	14	100%	Early feasibility studies
FORMA	Coaptation device	Axillary vein	20–24	89%	SPACER Trial
TriClip	Tricuspid edge to edge techniques	TF	24	100%	Triluminate
PASCAL	Tricuspid edge to edge techniques	TF	22	92%	Early feasibility studies warranted
Millipede System	Annuloplasty	S/TF		100%	FIH Study
PASTA device	Tricuspid edge to edge techniques	TJ/TA	8–12	90%	Early feasibility studies warranted
CAVI–TricValve	caval valve implantation	TF	27	100%	TRICAVAL

CAVI, caval valve implantation.

**Table 2:** Ideal and complicating factors for transcatheter tricuspid valve interventions^28 63^

Approach	Ideal factors	Complicating factors
Edge to edge repair	Coaptation defect<7 mm Adequate leaflet mobility Anteroseptal jet Confined leaflet prolapse or flail leaflet RV lead without primary leaflet obstruction	Gap>8.5 mm Thick/short leaflets Leaflet perforation Difficult echocardiographic visualisation CIED RV lead leaflet impingement Anteroposterior jet location Severe leaflet tethering
Annuloplasty	Annular dilation as principle mechanism of TR+central jet No or mild tethering (tethering height<0.76 cm, tenting area<1.63 cm^2^, tenting volume<2.3 mL) Adequate anatomic landing zone for anchor deployment	Severe annular dilatation exceeding device size Severe tethering (tethering height >1.0 cm, tenting volume>3.5 mL) Severe pulmonary hypertension CIED induced tricuspid regurgitation+impingement Anatomic proximity of RCA from annulus

CIED, cardiovascular implantable electronic device; RCA, right coronary artery; RV, right ventricle; TR, tricuspid regurgitation.

### Safety and outcomes

It was demonstrated that TTVI is a safe and effective treatment option for patients with symptomatic or severe TR who are not suitable for surgery.^30 31^ Baseline characteristics and procedural outcomes of transcatheter TR studies according to each device are presented in table 3. As a result of the demonstrated benefit and good safety profile, TTVI, for the first time ever, was mentioned in the 2021 European Society of Cardiology/European Association for Cardio-Thoracic Surgery Guidelines for the management of valvular heart disease as a therapeutic potion to treat, inoperable, anatomically eligible patients with significant TR.^32^

**Table 3:** Baseline characteristics and procedural outcomes of transcatheter tricuspid regurgitation studies according to each device^64^

	TriClip Nickenig *et al* (n=85)	PASCAL Fam *et al* (n=28)	FORMA Kodali *et al* (n=29)	CAVI Dreger *et al* (n=14)	Trialign Hahn *et al* (n=15)	Cardioband Nickenig *et al* (n=30)	TriCinch Denti (n=24)
Age, years	77.8±7.9	78±6	76±8	77 (68.2–82.0)	74±7	75±6.6	74±8
Female sex	66%	15 (54)	19 (66)	86%	13 (87)	22 (73)	20 (83)
Atrial fibrillation	92%	26 (93	24 (83)	na	10 (67)	28 (93)	n/a
NYHA III–IV	75%	28 (100)	25 (86)	86%	10 (67)	25 (83)	14 (58)
Transtricuspid CIED	14%	1 (3)	n/a	na	0 (0)	4 (13)	n/a
EuroSCORE II, %	8.7±10.7 (80)	6.2±5.2	8.4 5.3	14.6±11.6	n/a	4.1±2.8	5.5
**Echocardiography**							
LVEF, %	59.39±8.09 (73)	59±6	57±12	56.4±6.4	60±12	57±11	n/a
TAPSE, mm	1.44±0.31 (79)	16±3	14±0.4	16.1±5.2	16±4	n/a	n/a
Vena contracta width, mm	17.3 0.07	11.4±5	16±0.5	na	13±3	12.6±4.5	n/a
EROA, cm^2^	0.65 0.03	1.3±2.4	2.2±1.5	1.23±0.6	0.7±0.5	0.79±0.5	n/a
**Procedural and 30-day outcomes**							
Procedural success	na	24 (86)	27 (93)	100%	15 (100)	30 (100)	18 (81)
Procedural death	na	0 (0)	2 (7)	0	0 (0)	0 (0	n/a
Need for cardiac surgery	na	0 (0)	3 (10)	29%	0 (0)	0 (0)	n/a
30-day TR grade *>*moderate Follow-up	na	4/26 (15)	n/a	na	n/a	5 (24)	
Follow-up time, months	1 year	30 days	30 days	3	12 m	6	6
Mortality	7.1 (6/84)	2 (7)	2 (7)	8 (57%)	2 (13)	3 (10)	0 (0)
TR grade>moderate	na	4/26 (15)	2 (7)	na	n/a	5 (28)	(75)
NYHA I–II	83%	23/26 (88)	18/25 (72)	na	5/10 (50)	22 (88)	n/a

LVEF, left ventricular ejection fraction; TAPSE, tricuspid annular plane systolic excursion; TR, tricuspid regurgitation.

### Postprocedural antithrombotic therapy

TTVIs might pose an increased risk of thrombosis since the right side of the heart has a low-pressure circulation. However, the optimal antithrombotic therapy following transcatheter TV interventions remains controversial.^17 28^ Since, approximately 90% of patients undergoing TTVIs were known to have atrial fibrillation, they require systemic anticoagulation regardless of the procedure.^17^ In patients with no indication for systemic anticoagulation, they might benefit from antiplatelet therapy consisting of 4 weeks of aspirin plus clopidogrel, followed by aspirin daily for life.^28^

## Methods

### Search strategy

A systematic and comprehensive literature search was performed electronically using PubMed, Web of Science, Embase, OVID and Cochrane Library. Inclusion criteria were set to retrieve English studies that involved adult patients who underwent transcatheter intervention for TR. Studies were included if they detailed the main procedural steps of the percutaneous intervention being performed. The electronic search captured all articles till the date of 15 January 2021. Abstract-only papers were excluded. The search identified studies based on the following search terms: (Tricuspid regurgitation) OR (TR) OR (Tricuspid Valve Regurgitation) OR (Tricuspid Valve Incompetence) OR (tricuspid incompetence) OR (tricuspid valve insufficiency) OR (tricuspid insufficiency) AND (Percutaneous intervention) OR (Transcatheter intervention) OR (Coaptation device) OR (Annuloplasty device) OR (Heterotopic caval valve implantation) OR (CAVI) OR (Orthotropic transcatheter tricuspid valve replacement) OR (Transcatheter tricuspid valve intervention) OR (Transcatheter tricuspid valve repair) OR (Forma repair system) OR (TriClip) OR (MitraClip) OR (Trialign) OR (TriCinch) OR (Cardioband) OR (Minimally invasive annuloplasty) OR (MIA) OR (PASCAL system) AND (Mitral) NOT (Aortic) NOT (Bicuspid) NOT (Pulmonary).

### Screening

The electronic search of the databases and the hand search of the bibliographies yielded 803 articles. The two authors AM and VZ performed the title/abstract, full-text screening and data extraction. The discrepancies regarding the eligibility of the articles were resolved by a discussion among authors until a consensus was reached. After deduplication, 612 articles remained. Overall, 129 articles underwent full-text screening. The final count of articles included in the study was 17 studies.

### Data extraction

Data extraction was previously shared among authors to ensure consistency in pooling the data, captured information of the author, year of publication, the type of the study, the procedure being performed and the details of the procedural steps.

### Percutaneous interventions procedural steps

#### Coaptation devices

##### TriClip

##### Procedural video: Click Here

The TriClip System is an edge-to-edge repair technique that was developed and based on the MitraClip device. The technique has been used in the treatment of mitral regurgitation. This, as a result, has gained the technique a lot of experience in the field.^33^ Currently, the TriClip device has four different implant sizes that can be used.

The TriClip System configuration consists of two parts: The Clip Delivery System (CDS) and the Steerable Guide Catheter (SGC).^30^ The former includes an implantable clip with grippers, a delivery catheter and a steerable sleeve, which are used to manipulate and advance the clip, while the latter includes a dilator.^30^ Control knobs, levers and fasteners are used to steer and actuate the guide and CDS. The F/E knob flexes and extends the delivery catheter. The S/L knob, recently introduced with the G4 model, enables septal and lateral motion. The ± knob straightens and curves the guide, which adjusts the height of the system with respect to the valve.^30^ Withdrawing the device orients it posteriorly, advancing it orients it towards the aorta, clockwise rotation orients it towards the septum and anticlockwise laterally.^33^

Two methods using the TriClip System have been described: The Triple Orifice Technique, where the clips are placed centrally between the septal leaflet, anterior tricuspid leaflet, and the posterior tricuspid leaflet, and the bicuspidisation technique, where the clips are placed most of the time between the septal and anterior tricuspid leaflets. The bicuspidisation technique has been more feasible and done more frequently.^33^

The TriClip procedure is performed under general anaesthesia and with transesophageal echography (TEE) and fluoroscopy guidance.^34^

First, a right femoral venous access is obtained with a 24F sheath insertion. A double ProGlide preclosure is performed. Unfractionated heparin is administered to achieve an activated clotting time of 250–350 s^35^. Under TEE and a bicaval view in addition to fluoroscopy and a right anterior oblique (RAO) guidance, a stiff wire, is advanced to the superior vena cava (SVC). An SGC is then exchanged for the sheath and advanced over the wire with the ± knob turned 180° towards the ‘−’ to aid in straightening the tip to allow easier advancement.^35^ Occasionally, straddling the SVC might be needed.^34^

The next step is done under TEE bicaval view guidance and left anterior oblique (LAO) on Fluoroscopy. Unsheathe the device in the RA with the ± knob returned to the neutral position and the wire retracted into the device, such that a parallel orientation of the system with the septum is achieved. Next, flex the F/E knob to steer the tip of the device towards the TV. It is of utmost importance to have it orthogonal to the annulus of the valve.^33^We also turn the S/L knob towards ‘L’ in order to free the device from the septum.^34^ The device now is in the RA above the TV. At this point, open the device and examine the functionality of the grippers. Then, close and advance into the RV, which is simultaneously detected as crossing TV leaflets under fluoroscopic RAO projection and TEE guidance.^35^ At this point, the TEE transgastric short axis view, the RVOT outflow view and the four chamber view are used interchangeably along with LAO projection on fluoroscopy to optimise and fine tune the device location and coaxiality with the TV leaflets in preparation for grasping them.^35^ The transgastric view is the most helpful in locating the maximal regurgitation site, as well as the size of the gap. This aids in determining the best clipping location. The anterior and septal leaflets are the most commonly clipped. The device is opened, and fine manipulations are performed further to achieve maximal overlap of the TV leaflets and the clip arms. Leaflet grasping can be done by simultaneously grasping them. However, if the G4 model is used, there is also a possibility of independent grasping with seperate activation of each gripper that also further permits leaflet grasping optimization. When the device position and clocking are deemed optimal on TEE and fluoroscopy and appropriate leaflet overlap is ensured, a slight pull back is performed, and then leaflet grasping is done on RAO projection and TEE guidance.^35^

Success of the procedure is assessed by fluoroscopy and TEE. It is defined by a reduction in the regurgitation by one or more grades without creating a valvular stenosis, in addition to the safe implantation of the clip without partial leaflet detachment or device migration.^36^ Once sure of the success of the procedure, the clip is deployed, the system is removed and the femoral venous closure with ProGlide sutures is performed.

##### PASCAL

##### Procedural video: Click Here

The Edwards Pascal transcatheter mitral valve repair system is an edge-to-edge repair technique adopted from mitral regurgitation (MR) repair for the treatment of TR.^37^ This device has a hybrid design combining the MitraClip leaflet attachment system that constitutes paddles and clasps along with a central spacer resembling that of the FORMA device.^38^,^17 33^

The PASCAL System is made up of an implant that has a 10 mm central nitinol woven spacer. It behaves like a filler in the regurgitant orifice of the atrioventricular (AV) valve, along with two paddles and clasps that attach to the valve leaflets.^33^ The system also consists of a 22 French steerable guide sheath, a steerable catheter and an implant catheter with the implant preattached to its distal end. Rotational knobs on the external handles of the guide sheath and steerable catheter allow flexion movements. The handle of the implantation catheter allows for leaflet grasping simultaneously, or one clasp at a time.^39^ The spring-loaded paddles (25 mm width in grasping position) and clasps (10 mm length) are 1.5 times the width of the MitraClip System, which allows the distribution of the load across the surface area of the inserted leaflets. However, a careful evaluation of the subvalvular apparatus (papillary muscles, trabeculations or prominent moderator band) is usually needed to ensure successful device navigation (especially in non-dilated ventricles).^33 40^ The device can also be elongated to achieve a narrow profile and reduce the risk of entanglement.^39^

The procedure is done either in a catheterisation laboratory or a hybrid operating room under general anaesthesia with fluoroscopic and TEE guidance.^33^

The procedure starts by obtaining right femoral venous access and inserting a 22F guide sheath. The guide sheath is advanced all the way into the RA. After that, the introducer and wire are removed, along with air removal from the system. Then, the steerable and implant catheters are inserted into the guide sheath. They are advanced to the tip of the guide sheath until the implant is exposed in the RA. The implant clasps are closed, and the steerable guide flexed down by turning the knob so that the implant lies just above the TV in the RA. It is very important to ensure that the implant is perpendicular to the tricuspid plane. Then, the system is opened and advanced towards the ventricle to have one of the leaflets grasped by the first clasp on breath hold. When this is ensured, a gentle pull back of the implant is done to optimise the position of the first leaflet. The clasp is then closed and secured. The system is manipulated by the knobs along back-and-forth manoeuvres to swing the system to ensure a proper grasp over the second leaflet. Occasionally, both leaflets can be grasped simultaneously.^33^

Finally, when both leaflets are closed, the residual TR grade and the transvalvular gradient are evaluated before the device is deployed.^33^ If the result is suboptimal, the implant could be recaptured, repositioned or removed, if necessary. Finally, the implant is detached from by cutting the sutures attached to the clasps and pulling out the safety pin at the system and the latter is retracted, and conventional vascular closure is performed.^33 40^

##### FORMA

##### Procedural video: Click Here

The Edwards FORMA System is a combination of a spacer and a rail. The spacer is placed in the regurgitant orifice to reduce TR and to provide a surface for leaflet coaptation.^41^ The rail is anchored to the endocardial surface of the RV and over it the spacer is delivered.^41^ The FORMA device had favourable outcomes in patients with pacemakers or cardiac implantable electronic devices in situ.^42^,^17^

The procedure is done under general anaesthesia with fluoroscopic and transesophageal echocardiography (TEE) guidance in a Cath lab or hybrid operating room. Venous access to the left subclavian is achieved by surgical cut-down or percutaneously from the axillary vein. A 24F sheath is inserted and advanced to the level of the left innominate vein/SVC junction to support the delivery catheter. Then, a right ventriculography is done to identify a target location that allows the device to be perpendicular to the valve plane as well as to allow all leaflets to coapt with the device. After that, vascular closure is obtained with ProGlide (Abbott vascular) sutures or surgically.^41^

Through the sheath, a steerable delivery catheter is advanced and manipulated under TEE guidance, serves to station the anchor and rail in their correct position. The anchor target is on the lateral wall of the RV close to the interventricular septum and perpendicular to the annular plane to ensure optimal coaptation of the TV leaflets.^41^ To avoid entanglement in the valve chordae, a large balloon close to the tip of the delivery system is inflated prior to crossing the valve. A specially designed retrieval system allows the retrieval of the rail and spacer during all stages of the procedure if needed until the sheath is removed.^41 43^

The rail is anchored to the RV with six curved prongs designed in a manner that prevents it from exiting into the pericardial space while still grasping the RV myocardium.^41 43^ The spacer is slid to straddle the TA. The spacer is a foam-filled polymer balloon that passively expands via holes in the spacer shaft. It is round and tubular is shape, 42 mm long and has diameters of 12, 15 and 18 mm. The TR is improved as the spacer is positioned across the TA to provide the native leaflets with a new surface for coaptation. The spacer self-centres in the regurgitant orifice, and minor adjustments of the spacer location and along the rail improve coaptation of the leaflets if necessary. After confirming adequate reduction of TR with aid of echocardiography and haemodynamic monitoring, the position of the spacer is locked on the rail.^41^

Finally, the length of the rail is trimmed, and its proximal end is sutured to the subcutaneous tissue of the deltopectoral groove to fixate the rail and the spacer. Venous closure is then achieved either percutaneously or surgically.^41^

##### Annuloplasty devices

###### Trialign

###### Procedural video: Click Here

The Trialign device is a valve annuloplasty apparatus that is introduced transjugularly to reduce the tricuspid annular diameter via tissue plication.^44^,^45^

The goal of this procedure is to perform a transcatheter bicuspidisation of the TV. Two 14 French sheaths are placed in the ventral and lateral portions of the right internal jugular veins. In addition to that, a guide catheter via femoral access is placed in the right coronary artery.^46^

A deflectable tricuspid guide catheter is advanced via jugular access to the RV. The latter is designed to aid in directing procedural devices across the TV orifice and towards the TA. A tricuspid wire delivery catheter is then advanced to access the ventricular side of the TA at the site of pledget delivery and then, with the aid of fluoroscopy and TEE, it is advanced to the septoposterior location. Then a crossing wire is introduced into the delivery catheter and advanced to penetrate through the annulus from the ventricular side to the atrial side by that providing a path for pledget delivery. The crossing wire is mounted with a radiofrequency energy source at the distal tip that is activated as the wire is moved through the tricuspid annular tissue. Echography is then used to confirm the position of the crossing wire.^46^

Once the crossing wire is properly positioned in the septoposterior of the TA, an endovascular snare system is used to trap the distal tip of the crossing wise, which is retracted through the jugular access site. After snaring of the crossing wire, a tricuspid pledget deliver catheter is positioned in the RA facing the deflected tricuspid guide catheter positioned under the TA in the RV.^46^

The pledget delivery catheter is moved through the TA into the tricuspid wire delivery catheter until it seats the distal portion of the pledget on the ventricle side of the TA. Next, the pledget delivery catheter is retracted through the annulus to the atrial side where it deploys the proximal portion of the pledged. The steps are then repeated on the opposite anatomic site of the anteroposterior commissure to position the second pledget.^46^

A dedicated plication lock delivery catheter is advanced towards the annulus over the pledget sutures, which results in the plication of the annulus and the leaflet in between the two pledgets. A lock is then deployed onto the sutures. Finally, a suture cutter catheter is tracked over the sutures to lock the implant and cut the sutures above the lock, via a blade contained in the tip of the catheter, to allow the removal of the proximal suture parts.^46^

##### TriCinch

##### Procedural video: Click Here

The percutaneous TriCinch System is an annuloplasty device designed for the management of functional TR by means of a transfemoral fixation of a corkscrew into the anteroposterior TV annulus.^47–49^

The system consists of a Dacron band connecting both the self-expanding stent, and a corkscrew anchor.^48^ The stent is available in four different sizes (27, 32, 37 and 43 mm), to ensure proper engagement at the level of the inferior vena cava (IVC).^48^ The anchoring corkscrew remodels the AP annulus by pulling the system towards the IVC, while tension maintenance in ensured by fixation of the stent.^49^

The procedure is planned based on cardiac CT to identify the optimal implantation site and is performed under general anaesthesia with the guidance of fluoroscopy and echocardiography (TEE, transthoracic echocardiogram (TTE) or intracardiac echocardiography).^48 49^

Frist, a coronary wire may be placed as a marker in the right coronary artery.^49^ Then an 18F TriCinch delivery system is advanced into the RA and pointed towards the target site on the TA. The latter is done through a 24F Gore Dryseal sheath, which is introduced into the right femoral vein.^49^

Based on the baseline cardiac CT along with intraprocedural fluoroscopy, the implantation location is identified, and the stainless-steel corkscrew is fixed. The fixation is localised in the anteroposterior TV annulus.^48^ Then, right coronary angiography and an echocardiographic ‘pull test’ are performed to check for any interference with the corkscrew.^48^ Afterwards, the stent delivery system is brought forward and connected to the stainless-steel corkscrew via the Dacros Band.^48^ After system tensioning, septolateral dimension and the regurgitation grades are assessed and compared with baseline. After significant reductionof the tricuspid regurgitation is obtained, the stent is deployed to maintain the TV tension by deploying the sent subhepatically in the IVC.^48^

##### Cardioband

##### Procedural videos: click here: Video 1, Video 2

The Cardioband Tricuspid System targets the management of patients with TR with annular dilation by annular reduction using an adjustable implant.^50 51^

The Cardioband System consists of four main accessories which are: (1) the implant (a polyester sleeve with radiopaque markers that has a contraction wire running in the sleeve and connected to an adjustment spool which adjusts the length of the implanted device), (2) the TF Delivery System (TDS) (consisting of an SGC, an implant catheter with the Cardioband implant mounted on its distal end and a 24F steerable sheath), (3) the implantable mental anchors and anchor delivery shafts and finally (4) the size adjustment tool (SAT).^50^

The procedure is similar to that of mitral valve treatment, with a major difference with the band clockwise implantation from the anteroseptal commissure to the septal annulus first part, after the coronary sinus.^52^

First, transfemoral artery access is established so that right coronary artery angiography is performed, and a wire is placed to mark it and prevent any compression or kinking.^48^

Then, transfemoral venous access is established and a 24F sheath is inserted. Through which a highly manoeuvrable TDS is advanced into the RA under TEE guidance and directed towards the TA. The implant is deployed and fixed by inserting up to 17 anchors on the atrial surface of the annulus.^50^ The implantation process is initiated on the antero-septal commissure, continues clockwise to end after the coronary sinus and the postero-septal commissure.^50^

After deploying the anchors, the implant catheter is exchanged for the SAT, which is advanced over the contraction wire to reshape of the annulus. Then, the band is clinched, by that reducing the septolateral TA and anteroposterior TA diameters.^50^ If needed, implant size can be adjusted and guided by fluoroscopic and echocardiographic imaging.^50^ Finally, percutaneous closure of the access sites is performed.^52^

##### Transcatheter CAVI

Transcatheter CAVI has been used to treat severe TR.^53^ This procedure can be done using a balloon expandable valve (BEV) or a self-expandable TricValve.^54 55^

Patients undergo screening for anatomic suitability via a computed tomographic angiogram. This studies the anatomy and diameter of the device landing zones in the SVC and IVC at the cavoatrial junction. Right heart catheterisation is done to confirm severe TR and caval backflow. Patients with severe pulmonary hypertension and TAPSE less than 10 are excluded. The procedure is performed under general anaesthesia along with fluoroscopic, transesophageal echo and haemodynamic guidance.^53^ Unfractionated heparin is administered to reach an activating clotting time above 250 s throughout the procedure.^53^ After the procedure is performed, venous closure is achieved by either a so-called Z-suture of the skin or the use of percutaneous closure devices.^53^ After a short stay at the hospital, all patients are discharged on vitamin K antagonists.

##### CAVI using BEV

There is a growing experience in the off-label usage of the Edwards Sapien XT and Sapien three valves for treating severe TR. Both were previously commercially used for the treatment of aortic stenosis.^53^ This procedure requires the implantation of a self-expandable stent. This stent serves as a landing zone for the BEV to facilitate its fixation. This is done because the cavoatrial junction anatomy in the setting of severe TR precludes direct implantation of a BEV. Classically, one BEV is implanted over a self-expandable stent in the IVC. However, a bicaval valve implantation (BiCAVI) procedure is also possible, where in addition to the IVC, another BEV is implanted on a self-expandable stent in the long segment of the SVC.^49^

First, the right femoral vein is punctured and a 6F sheath is placed in it. A stiff wire is advanced to the SVC, and then a 16F or 20F sheath is advanced to reach a level just below the diaphragm.^53^ Then, a self-expandable stent that is tailored to the IVC diameter (eg, 30×80 mm) is implanted in the IVC at the level of the diaphragm and protrudes around 5 mm into the RA.^53^ A 29 mm BEV is mounted on the delivery system and deployed inside the stent at the level of the diaphragm with its lower edge directly superior to the confluence of the first hepatic vein, which is marked with a right Judkins catheter. If a BiCAVI procedure is to be done, an additional self-expanding stent is deployed in the SVC above the RA inflow to avoid the risk of vessel wall damage and rupture, and the SVC prosthesis is implanted in the same manner.^53^

##### CAVI using the self-expandable TricValve

##### Procedural videos: click here: Video 1, Video 2

The TricValve System constitutes two self-expandable valves designed specifically for SVC and IVC implantation in the low-pressure circulation. The SVC valve is a belly-shaped tapered device designed to anchor in a dilated, tapered SVC configuration.^53^ The IVC valve, on the other hand, is deployed at the level of the diaphragm and protrudes into the RA. While, both devices are made from bovine pericardium, the inner part of the atrial stent portion is lined with a polytetrafluorethylene skirt.^53^

All procedures are fluoroscopy and transesophageal echocardiography guided.^53^

To implant the valve, access is obtained via the right femoral, left femoral vein and left basilic veins. Then, an Amplatz wire with a long soft J-tip is inserted and advanced through the right femoral vein to the RA and into the right internal jugular vein. To note that both devices are loaded into 27F catheters and undergo sheathless implantation.^53^ First, the SVC valve implantation process is performed. A catheter is advanced to the right pulmonary artery to mark the IVC–right pulmonary artery crossing. A Lunderuist guidewire is placed to mark the superior border of the RA. Then, SVC angiography is performed.^53^

Then, the SVC valve is advanced and deployed with the landing zone of the enlarged midportion of the stent (Valve Belly) above the marked right pulmonary artery. The upper edge of the valve should avoid the occluding the brachiocephalic vein, which can be marked by a multipurpose wire. The lower edge of the valve should be at the atrium–SVC junction with a small protrusion into the RA tolerated. Then, deploy the IVC valve. Hepatic vein angiography and Lunderuist guidewire can be used to identify the hepatic vasculature and the RA, respectively. With the upper, skirt-lined segment of the stent protruding into the RA, and the device fully anchored in the IVC, the IVC valve is deployed as well.^53^ The constrained segment of the stent should be aligned with the cavoatrial junction while carefully pulling back the catheter to avoid occlusion of the hepatic vein inflow just below the diaphragm.^53^ Thus, a safety margin of 5 mm should be maintained to avoid a low or high valve position, which might lead to hepatic vein obstruction or paravalvular regurgitation, respectively.

#### Investigational techniques

##### TRAIPTA

The transatrial intrapericardial tricuspids annuloplasty TRAIPTA is a concept where after establishing venous access, pericardial access is obtained by puncturing the right atrial appendage. This technique allows the delivery of an implant along the AV groove within the pericardial space that exerts compressive forces over the annulus. Interactive adjustment of the implant controls its tension and helps modifying and reducing the TR. The right atrial puncture is sealed at the end of the procedure with a nitinol device. While the procedure has been performed in pigs, human testing with a new device is under development. Limitations of such a procedure include the need for a free pericardial space, while the risk of coronary artery compression remains a risk.^56–58^

##### Millipede System

The Millipede System, a complete repositionable and retrievable ring, can be implanted both surgically and via transcatheter, on the right atrial side, to restore the shape of the TV. While this technique has been used for the management of mitral valve pathologies, the use of the Millipede System for TR is promising.^58^

##### Transcatheter TV replacement

Transcatheter TV replacement whether after tricuspid annuloplasty (valve in ring) or a degenerated surgical bioprosthesis (valve in valve) is also under study. The NaviGate self-expanding tricuspid AV valved stent has been studied in a preclinical pig model and used in humans under the title of compassionate use. The device comes in different sizes and constitutes a ninitol tapered stent with three xerogeneric pericardial leaflets and annular winglets that allow for annular and leaflet fixation without protrusion of the stent into the surrounding chambers.^58^

##### PASTA

Finally, the percutaneous pledget-assisted suture tricuspid annuloplasty targets the reduction of the size of the TV orifice by approximating the septal and lateral tricuspid annuli. This is done via pledgeted sutures between the middle of the anterior and the posterior lead. This technique has proven feasible, and results in a reduction in the annular area along with TR in swine models. PASTA looks promising for patients with TR without other good treatment options.^59^

## Conclusion

TR is a highly prevalent disease that is associated with high mortality and morbidity when left untreated. Historically, surgical repair or replacement were the only therapeutic options; however, due to their high complication rate they were often deferred. TTVIs that have been showed to be feasible, associated with low mortality rates, and effective in reducing TR associated with alleviation of symptoms.^60^ In this article, we presented a technical review with a detailed step-by-step procedural approach of the different TTVIs for the treatment of TR along with video guidance.

## Data Availability

No data available.
